# Supplementary material for: Biochemical Profile and In Vitro Therapeutic Properties of Two Euhalophytes, Halocnemum strobilaceum Pall. and Suaeda fruticosa (L.) Forske., Grown in the Sabkha Ecosystem in the Algerian Sahara
Source: Molecules. 2023 Apr 19;28(8):3580. doi: 10.3390/molecules28083580 (PMC10141351; doi:10.3390/molecules28083580)
Supplement: Supplementary file 1 [file molecules-28-03580-s001.zip › molecules-2322542-supplementary.pdf]

molecules

Article

Biochemical Profile and *In Vitro* Therapeutic Properties of Two Euhalophytes, *Halocnemum strobilaceum* Pall. And *Suaeda fruticosa* (L.) Forske., Grown in the Sabkha Ecosystem in the Algerian Sahara

Noura Gheraissa[1,2], Ahmed Elkhalifa Chemsa[1,3], Nezar Cherrada[1,2], Ebru Erol[4], Eman Ramadan Elsharkawy[5], Djilani Ghemam Amara[3,6], Soumeia Zeghoud[7] Abdelkrim Rebiai[7,*], Mohammed Messaoudi[7,8], Barbara Sawicka [9], Maria Atanassova [9] and Maged S. Abdel-Kader[11,12*]

1. Laboratory of Biodiversity and Application of Biotechnology in Agriculture, El Oued University,El Oued 39000, Algeria
2 Department of Cellular and Molecular Biology, Faculty of Natural Science and Life, El Oued University, El Oued 39000, Algeria
3 Department of Biology, Faculty of Natural Science and Life, El Oued University, El Oued 39000, Algeria
4 Department of Analytical Chemistry, Faculty of Pharmacy, Bezmialem Vakif University, İstanbul 34093, Türkiye;
5 Department of Chemistry, Faculty of Science, Northern Border University, Arar 73213, Saudi Arabia
6 Chemistry Department, Faculty of Exact Sciences, University of El Oued, El Oued 39000, Algeria
7 Laboratory of Biology, Environment and Health, El Oued University, El Oued 39000, Algeria
8 Nuclear Research Centre of Birine, Ain Oussera, Djelfa 17200, Algeria
9 Department of Plant Production Technology and Commodities Science, University of Life Science in Lublin, Akademicka 15 Str., 20-950 Lublin, Poland;
10 Nutritional Scientific Consulting, Chemical Engineering, University of Chemical Technology and Metalurgy, 1734 Sofia, Bulgaria
11 Department of Pharmacognosy, College of Pharmacy, Prince Sattam Bin Abdulaziz University, Al-Kharj 11942, Saudi Arabia
12 Department of Pharmacognosy, Faculty of Pharmacy, Alexandria University, Alexandria 21215, Egypt
* Correspondence: rebiai-abdelkrim@univ-eloued.dz (A.R.); mpharm101@hotmail.com (M.S.A.-K.)

Table S1. Determination of phenolic compounds of some plants of the *Chenopodiaceae* family using HPLC analysis

Plant species	Phenolic compounds	Amount (µg/100 mg ED)	Reference
Suaeda fruticosa	Gallic acid	0.449±0.02	[12]
	Catechin	166.7±8	
	Chlorogenic acid	126.8±9	
	Caffeic acid	383±1	
	Quercetin	0.247±1	
	Kaempferol	176±1	
	Chlorogenic acid	288	[34]
	Caffiec acid	47	
	p-Coumaric acid	133	
	Gallic acid	51	
	Rutin	32	
	Vanillin	17	
	Vanillic acid	288	

***Beta vulgaris L.* (root)**	Gallic acid	11.01		[35]
	Catechol	7.38		
	p-Comuaric acid	0.74		
	Ferulic acid	0.68		
	o-Coumaric acid	1.31		
	Cinnamic acid	0.60		
	Myricetin	19.25		
	Neringenin	19.92		
	Kaempferol	3.02		
	Apigenin	2.65		
***Beta vulgaris L.* (root)**	Coumarin acid	325.0395	(ppm)	[36]
	Resorcinol	0.0562		
	Quercetin	136.0987		
	Kaempferol	43.2809		
	Naphthaline	0.1974		

References

12. Qasim, M., et al., *Antioxidant properties, phenolic composition, bioactive compounds and nutritive value of medicinal halophytes commonly used as herbal teas.* South African Journal of Botany, 2017. **110**: p. 240-250.

34. Gheraissa, N., et al., *Phenolic compound profile, and evaluation of biological properties of Bassia muricata (L.) Asch. aerial part.* International Journal of Secondary Metabolite, 2022. **Vol. 9, No. 3**: p. 335-347.

35. El-Beltagi, H., et al., *Evaluation of some chemical constituents, antioxidant, antibacterial and anticancer activities of beta Vulgaris L. Root.* Fresenius Environmental Bulletin, 2018. **27**: p. 6369-6378.

36. Shalaby, H. and A. Hassenin, *Effects of Fortification Stirred Yoghurt with Red Beet Powder (RBP) on Hypercholesterolemia Rats.* European Journal of Agriculture and Food Sciences, 2020. **2**.

.
